# Promoting stroke awareness through short movies and film festivals

**DOI:** 10.1111/cns.13710

**Published:** 2021-07-26

**Authors:** Jing Zhao, Renyu Liu

**Affiliations:** ^1^ Department of Neurology Minhang Hospital Fudan University Shanghai China; ^2^ Department of Anesthesiology and Critical Care Perelman School of Medicine at the University of Pennsylvania Philadelphia Pennsylvania USA

**Keywords:** movie festival, short movies, stroke, stroke awareness

*Wakeup Stroke 120*, our newly produced short movie, received four nominations at the 2021 New York City international film Festival (NYCIFF). These nominations include best short film, best short film leading actor, best short film director, and humanitarian awards. We won the humanitarian award and the best short film (Figure 1). This triggered a wave of news reports both in China and in the United States in May 2021.^1^, ^2^ Such news reports help us in promoting stroke awareness tremendously. May is the Stroke Awareness Month in the United States. The timing was perfect to convey such an important message using the tool of a movie. The film received very positive reviews and testimonials from the international community (Supplemental File 1). Through such multidisciplinary collaborations using short videos and short movies, we can potentially penetrate various barriers for popular science education and reach large audiences through various media platforms and news reports. This award‐winning short movie is highlighted on the home page of the NYCIFF (www.nyciff.com) along with our stroke awareness appeal speech.

**FIGURE 1:**
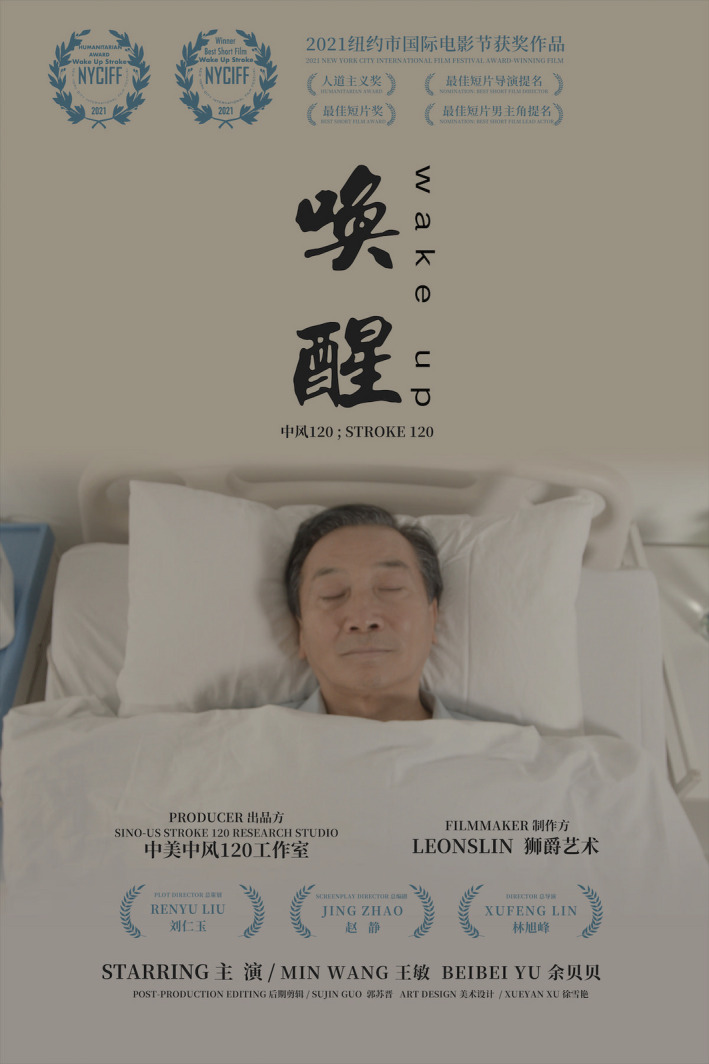
The movie poster for the Wakeup: Stroke 120. The movie can be viewed on YouTube: https://www.youtube.com/watch?v=ohNZYlyoTaA

This movie focuses on elderly people living alone who have a high risk of stroke, a growing issue globally for any of the aging societies. The aims of this movie are to wake up not only potential stroke patients, but also the societies to raise awareness for stroke and the elderly who live alone, thus being part of the community's efforts to prevent stroke‐related disasters. This movie is adapted from a true story, a story commonly seen in clinical practice nowadays. The movie tells the story of an elderly man whose wife has passed away and his only daughter is married to a man who lives in another country. While his daughter brought him to live with her family, he was not used to live abroad and felt lonely and decided to go home and lived alone in his old familiar community. While he is returning from a grocery store, he has a sudden onset of stroke. His grocery bag drops to the ground due to arm weakness. A young man passed by and noticed that the man might need help. Unfortunately, neither the elderly man nor the young man realized that the elderly gentleman was having a stroke despite his slurred speech, a sign that is critical for stroke recognition especially during the COVID‐19 pandemic.^3^ The elderly man thought of texting his daughter but changed his mind because he did not want to bother her, and went home. He lost consciousness at home and was not found until the next morning by neighbors. He was paralyzed and aphasic. His daughter rushed back home from abroad, but nothing could be done to reverse the tragic course of her father's stroke. At the end of the movie, we continue the education with a critical message for the public: Stroke rescue is a race against time. It is critical to send stroke patients to the nearest stroke center within the first three “golden hours,” since the outcome varies greatly and becomes more catastrophic if any delay occurs. If the elderly recognize the early symptoms of stroke, seek rescue help immediately, and are sent to a nearby hospital for immediate treatment, there is a much greater chance of a better outcome. Children, no matter how busy they are, should call their (elderly) parents often, and pay attention to their parent's health. If each of us acts like a stroke 120 volunteer (stroke awareness campaign volunteer) and offers fast and effective rescue by calling the medical emergency phone number immediately, then most of our stroke patients can return to a normal, happy, and long life. To prevent stroke and to provide rescue from stroke for the elderly living alone, the support of the whole community is needed in addition to care from their children. By establishing daily home visits and early warning systems for stroke, while learning to identify stroke in a timely fashion and the need for immediate rescue, many tragedies can be prevented.

In 2019, we focused our education effort on strokes occurring in the younger population to convey the message that stroke can occur at any age.^4^ Based on a true story, we produced a short movie titled “*love and rescue around you*.”^5^ This short movie tells the story of a businessman who has hypertension and works very hard. One night, he worked very late and had a sudden onset of stroke. Both he and his wife did not recognize the signs and symptoms of stroke, as he thought that he was just too tired. He asked his wife to help him go to bed hoping that he would feel better the next day. Unfortunately, his wife found him unresponsive the next morning and he died despite being sent to the hospital by the emergency medical service. A comparative story ending strategy was used in this short movie. In the second ending, the young businessman is rescued when his wife immediately recognizes that her husband needs immediate medical attention and calls the medical emergency phone number. The young man survives and is there for his wife and child. A narrative statement was highlighted at the end of the short movie: Stroke is not a disease specific for old people. It can occur at any age, even in children. It would be great if we educate young people about stroke signs and symptoms. As Dr. Anthony Rudd, from Kings College London, stated in the movie: “The important thing we need to do is to educate the general public about how to prevent stroke. Too often, we are leaving prevention far too late. We need to get the idea across that causes of stroke start very early on. If we can identify high blood pressure, identify high cholesterol and identify diabetes early on when people are in their teens, 20s, and 30s, then we can prevent strokes.” This movie was premiered in a movie theater in Shanghai, and this theater provided a big advertisement panel outside of the theater to promote our stroke awareness program free of charge for one year. The movie was later selected by the Shanghai Charity Short Movie Festival in 2020 with an honorary mention as one of the outstanding charity short movies.

Stroke awareness remains suboptimal across the world, even in well‐developed countries, and especially in young people. Based on a recent study, 2.7% of young adults, representing 2.9 million people in the United States, did not know any stroke symptoms.^6^ Short movies can potentially be used to educate young people and improve stroke awareness. The ongoing COVID‐19 pandemic has generated a profound global impact on stroke care.^7^ The education of stroke awareness continues but is interrupted significantly due to the pandemic.^8^, ^9^ Using short movies with touching stories can be a powerful tool to improve stroke awareness during the pandemic, since many people are staying at home due to social distancing.

We believe that short videos are very powerful tools for popular science educational purposes in the modern society. Our experiences have proven that the use of short videos is an effective strategy. Immediately after we kicked off our stroke awareness program using stroke 120 at the end of 2016 in China, we produced and released a one‐minute short animated video introducing stroke 120.^10^, ^11^ This video was well accepted throughout China. It has been translated into 33 local dialects. It is also broadcast through national and local TV stations, TV monitors in city buses, subways, and TV networks in hospitals.^12^ We subsequently produced 7 short animations targeting immediate stroke recognition and actions. Examples of subjects include *atrial fibrillation and stroke*, *pitfalls in stroke recognition and action*, *stroke awareness in children*, *stroke in the perioperative care*, *and stroke awareness for deaf individuals* (sign language version). The delay in recognizing stroke continues to be a global issue.^13^ In the future, tools for stroke early recognition, detection, and prediction will help to improve overall stroke care quality.^14^, ^15^

## CONFLICT OF INTEREST

None.

## Supporting information

Supplement File S1

## Data Availability

The data are available by sending email request to Dr. Renyu Liu: RenYu.Liu@pennmedicine.upenn.edu
