# Supplementary material for: Promoting stroke awareness through short movies and film festivals
Source: CNS Neurosci Ther. 2021 Jul 26;27(9):991–3. doi: 10.1111/cns.13710 (PMC8339529; doi:10.1111/cns.13710)
Supplement: Supplementary file 1 — Supplement File S1 [file CNS-27-991-s001.pdf]

**Supplemental file 1**

**Selected Comments for the Wakeup Stroke-120 movie**

1. The film is great, is very touching, brought tears to my eyes because my brother died of stroke. He was 51 years old.
   Roberto Rizzo (New York, USA)
   电影很棒， 很感人。我看哭了， 因为我哥哥死于中风。他才 51 岁。
   罗伯托·里佐（美国纽约）
2. I was watching with tears because it recalled my experience when a parent was sent to the emergency room while I was living abroad and I felt so helpless. This script is so true and heart touching. It also emphasizes how important the community role is and how critical for people to get involved to help early.
   Nan Lin (Beijing, China)
   我泪流满面地看完，因为它回忆起我在国外生活时，父母被送到急诊室的经历，我感到很无助。这个剧本真切切切。它还强调了社区角色的重要性，以及人们尽早参与帮助的重要性。
   林楠（中国北京）

3. I love the movie. It means so much to me because it happened to my mother. The geographical barriers made it impossible for her to access rapid stroke care. It was over ten years ago. I wish we knew better then.
   Chanannait Paisansathan (Chicago, USA)
   我喜欢这部电影。这对我意义很大，因为它发生在我母亲身上。地理障碍使她无法获得快速中风救治。 那是十多年前的事了，我希望我们那时知道得更多。
   查纳奈特 • 派桑萨坦（美国芝加哥）

4. It is so professional and has such a powerful story!
   Anthony Rudd (London, UK)
   它是如此专业， 有这样一个感染力的故事！
   安东尼 • 陆克文（英国伦敦）

5. The very first thing I did after seeing the movie was forward it to my father! As a physician it’s a common story that I encounter these days. It was beautiful to see it as a well-taken short film. Every society across the globe in the 21st century can relate to this story. The cinematography was beautiful. The actors did an excellent job. Inspiring.
   Siju V Abraham (Kerala, India)
   看完电影后我做的第一件事就是把它转发给父亲！ 作为一名医生，这是我临床上常见的故事。把它拍成一部很好的短片，真美。21 世纪全球每个社会都可以与这个故事联系起来。电影摄影很美。演员们干得非常出色。鼓舞人心的。
   西朱五世亚伯拉罕（印度喀拉拉邦）

6. The film is very profound, moving, and beautiful. I love the music - and such an important issue, so well highlighted here.
   Lotje Sodderland (London, UK)
   这部电影非常深刻，感人，美丽。我喜欢它的音乐 – 把一个重要的问题如此巧妙地突出出来。
   洛杰 • 索德兰（英国伦敦）

7. The most important thing is this film will save lives
   Xuesheng Liu (Anhui, China)
   最重要的是这部电影将拯救生命
   刘学胜（中国安徽）

8. The perfect mingle of medicine and art
   The Youth Daily (Shanghai, China)
   (http://wx.youthdaily.cn/index.php/Html/preview?id=16208976071148593647）
   医学与艺术的完美交融
   《青年报》（中国上海）

9. This is a dramatic film with clear message.
   Grethe Andersen (Aarhus, Denmark)
   这是一部具有明确信息的戏剧性电影。
   格雷斯・安徒生（丹麦奥胡斯）

10. The movie is wonderful and thought provoking!
    Uchunor Michael (Lagos, Nigeria)
    这部电影很棒， 发人深省!
    乌丘诺・迈克尔（尼日利亚拉各斯）

11. Beautiful film that is very impactful!
    Veena Graff (Philadelphia, USA)
    非常有影响力的美丽电影!
    维娜・格拉夫（美国费城）

12. A very important work! Community education is critical for stroke immediate recognition. We have to race with time to save lives.
    Xiang Qian (San Francisco, USA)
    一项非常重要的工作！社区教育对于中风的即时识别至关重要。我们必须与时间赛跑来拯救生命。
    钱湘（美国旧金山）

13. Wake up public awareness for elderly living alone! This movie is hot
    Labor Newspaper (Shanghai, China)
    (https://www.51ldb.com/shsldb/wt/content/017964b3dd8cc00100005499e74cbcbc.htm)
    唤醒公众对独居老人的认识！这部电影很火爆
    劳动报（中国上海）

14. It's a really great movie! Not only it is very touching, it also delivers practical information to the people, and it truly combines medicine with art, popular science and education!
Yanqin Lou (Irvine, USA)
这是一部很棒的电影！它不仅很感人，还为人民群众提供实用信息，真正把医学与艺术、科普、教育结合起来！
楼燕琴（美国欧文）

15. Perfect combination of medicine and art
Dandan Wang (Nanking, China)
医学与艺术的完美结合
王丹丹（中国南京）

16. Worth spreading widely
Zezong Gu (Missouri, USA)
值得广泛传播
顾泽宗（美国密苏里州）

17. Well-deserved for the awards!
Weiren Sun (Taiwan)
获奖当之无愧！
孙伟仁（台湾）

18. Great short movie, especially the summary at the end of the movie.
Hang Zhao (Newark, USA)
伟大的短片， 尤其是电影结尾的总结。
赵航（美国纽瓦克）

19. Powerful.
Scott Kasner (Philadelphia, USA)
有冲击力（强大）！
斯科特•卡斯纳（美国费城）

20. It is great, and I'm sure it proves very helpful for stroke awareness. It would be wonderful to have something like that over here especially in the more underserved communities.
Annie Jones (Philadelphia, USA)
这很棒， 我敢肯定， 它被证明对提高中风意识是非常有帮助。在美国， 特别是在服务不足的社区， 要是有这样的东西真是太好了。
安妮•琼斯（美国费城）

21. A short movie with a perfect mingle of medicine and film art. It allowed the audience to learn stroke rescue knowledges while enjoying the movie.
    Guogen Sun (Shanghai, China)
    一部将医学和电影艺术完美结合的短片。它允许观众在欣赏电影的同时学习中风救援知识。
    孙国根（中国上海）

22. Short and refined
    Guozhang (Gary) Cheng（Baltimore, USA）
    短而精致
    程国章（加里）（美国巴尔的摩）

23. A pleasure to watch with an important message.
    Dennis Harris (Philadelphia, USA)
    很高兴（通过电影）看到如此重要的信息。
    丹尼斯 • 哈里斯（美国费城）

24. It's an incredible way of creating awareness on stroke! It's short and crisp, yet a powerful story that would help a lay bystander to diagnose the problem and help the elderly!
    Girija Prasad Rath (New Delhi, India)
    这是一个用令人难以置信的方式来提高中风意识！简短和清晰， 有一个强大的故事， 这将有助于一个外行旁观者识别中风， 并帮助老年人！
    吉里贾 • 普拉萨德 • 拉斯（印度新德里）

25. You’re going to need tissues.
    Laura Brzyski (Philadelphia Magazine, Philadelphia, USA)
    你需要准备纸巾（擦眼泪）。
    劳拉 • 布日斯基（美国费城费城杂志）
